# Prenatal Lead and Depression Exposures Jointly Influence Birth Outcomes and *NR3C1* DNA Methylation

**DOI:** 10.3390/ijerph182212169

**Published:** 2021-11-19

**Authors:** Allison A. Appleton, Kevin C. Kiley, Lawrence M. Schell, Elizabeth A. Holdsworth, Anuoluwapo Akinsanya, Catherine Beecher

**Affiliations:** 1 Department of Epidemiology and Biostatistics, School of Public Health, University at Albany, 1 University Place, Rensselaer, NY 12144, USA; lmschell@albany.edu; 2 Department of Obstetrics and Gynecology, Albany Medical College, 391 Myrtle Avenue, Albany, NY 12208, USA; kckiley@aol.com; 3 Department of Anthropology, College of Arts and Sciences, University at Albany, 1400 Washington Avenue, Albany, NY 12206, USA; 4 Department of Anthropology, Washington State University, P.O. Box 644910, Pullman, WA 99164, USA; e.holdsworth@wsu.edu; 5 Bon Secours, Obstetrics and Gynecology, 210 Medical Park Boulevard, Petersburg, VA 23803, USA; anusobanjo@gmail.com; 6 Three Village Women’s Health, 100 S Jersey Avenue, Setauket, NY 11733, USA; cabradsh@gmail.com

**Keywords:** lead, depression, birth outcomes, *NR3C1*, epigenetics, developmental origins of health and disease

## Abstract

Many gestational exposures influence birth outcomes, yet the joint contribution of toxicant and psychosocial factors is understudied. Moreover, associated gestational epigenetic mechanisms are unknown. Lead (Pb) and depression independently influence birth outcomes and offspring *NR3C1* (glucocorticoid receptor) DNA methylation. We hypothesized that gestational Pb and depression would jointly influence birth outcomes and *NR3C1* methylation. Pregnancy exposure information, DNA methylation, and birth outcome data were collected prospectively from *n* = 272 mother–infant pairs. Factor analysis was used to reduce the dimensionality of *NR3C1*. Multivariable linear regressions tested for interaction effects between gestational Pb and depression exposures with birth outcomes and *NR3C1*. Interaction effects indicated that higher levels of Pb and depression jointly contributed to earlier gestations, smaller infant size at birth, and asymmetric fetal growth. Pb and depression were also jointly associated with the two primary factor scores explaining the most variability in *NR3C1* methylation; *NR3C1* scores were associated with some infant outcomes, including gestational age and asymmetric fetal growth. Pb and depression can cumulatively influence birth outcomes and epigenetic mechanisms, which may lay the foundation for later health risk. As toxicants and social adversities commonly co-occur, research should consider the life course consequences of these interconnected exposures.

## 1. Introduction

Gestation is a critical period of human development whereby adverse exposures can affect developing body systems and influence health over the life course [1,2]. A myriad of gestational exposures, including environmental toxicants and psychosocial adversities, can independently influence fetal development and birth outcomes, with enduring growth and neurodevelopmental effects identifiable years later [3,4,5,6]. While illustrative of the life course health impacts of gestational exposures, this area of research typically focuses on identifying or isolating the effects of single exposures on health, with less attention paid to considering the joint effects of experiencing multiple exposures simultaneously. Social and environmental risks do not exist in isolation but instead co-occur and likely work synergistically to affect health outcomes [7]. In order to more accurately quantify the impact of gestational exposures, an interdisciplinary approach is needed that focuses on the broad range of factors within the environment. Doing so will not only increase the precision of our effect estimates and enhance causal inferences, but will also help improve clinical and policy recommendations for pregnant women.

The deleterious impacts of gestational exposure to heavy metals have been well documented [8]. Lead (Pb) in particular has received much attention given its neurotoxicity and widespread presence in the environment [9,10]. There is no known safe level of Pb exposure, and Pb can have harmful effects even at low levels, particularly for developing fetuses [9,10]. Maternal blood Pb can cross the placenta where it accumulates causing reduced nutrient transfer, oxidative stress, and abnormal function [9,11]. Moreover, the fetus’ small body size, rapid growth, and limited ability to eliminate harmful substances in utero can increase vulnerability to Pb exposure [10]. Several studies have found that higher levels of prenatal Pb exposure was associated with lower birth weight [9,10,11,12,13,14], reduced birth length [9,10,13], and/or reduced head or chest circumference [9,13,15]. While some studies have observed a null association [16,17,18], the accumulated evidence suggests gestational Pb exposure is an important risk factor for birth outcomes. Likewise, maternal depression is an important perinatal risk factor because of its potential to contribute to deleterious outcomes for both the mother and infant, and its relative high prevalence in the population [19]. Several studies have shown maternal depression during pregnancy is associated with smaller birth size outcomes and preterm births [20]. For example, a recent meta-analysis of 29 studies found that prenatal depression was associated with a nearly 50% increased risk of having a low birth weight infant [21]. Given the similar patterning in the associations between prenatal depression and Pb exposures and birth outcomes, it is plausible that Pb and depression exposures may work together during pregnancy and jointly exacerbate risk. This hypothesis has not yet been tested, though related work suggests this synergy is possible. For example, a recent review summarized research that has tested a joint contribution hypothesis for pediatric health outcomes and found that parental social stress consistently exacerbated the deleterious effects of prenatal air pollution exposures for birth outcomes [22]. In the present study, we extend this line of inquiry and focus specifically on whether gestational Pb and depression exposures can similarly and jointly affect birth outcomes.

The biologic mechanisms linking prenatal toxicant and psychosocial exposures are not fully understood. Accumulating evidence suggests that epigenetic alterations may link gestational exposures to child health [23,24,25,26]. Epigenetics involves altering patterns of gene expression without modifying the underlying nucleotide sequence of DNA. Epigenetic mechanisms, such as DNA methylation, can be altered during gestation and remain relatively stable over time to potentially exert long-term impacts on health and physiologic processes of the offspring [6]. DNA methylation to genes involved in the hypothalamic–pituitary–adrenal (HPA) axis has been the focus of much gestational programming research [27,28,29]. The HPA axis is the major neuroendocrine system that regulates body systems and responses to stress, including the regulation of glucocorticoids through the actions of the glucocorticoid receptor (NR3C1). Several studies have shown gestational *NR3C1* methylation is associated with birth outcomes and neurodevelopmental problems in offspring [30,31,32,33]. Separate sets of studies have also shown that *NR3C1* methylation is sensitive to gestational depression and metals exposures. For example, one prospective study among *n* = 222 mother and infant pairs found that higher prenatal Pb exposure, alone and in combination with other heavy metals and essential elements, was associated with increased placental *NR3C1* methylation [34]. Similarly, several studies have shown maternal depression during pregnancy to be prospectively associated with gestational *NR3C1* methylation [30,31,32], with a recent meta-analysis (*n* = 977 individuals) suggesting a small positive association between prenatal psychosocial adversity and *NR3C1* methylation [35]. Despite these parallel sets of studies, researchers have not considered gestational Pb and depression exposures as jointly modulating *NR3C1* methylation.

In this study, we examined the joint contribution of Pb and depression occurring during gestation in relation to birth outcomes, and considered *NR3C1* methylation as a potential shared biological mechanism that may help explain how these environmental and psychosocial factors influence infant health. We hypothesize that (1) depression during pregnancy and prenatal Pb exposure will jointly contribute to worse infant outcomes than either exposure independently, (2) gestational Pb and depression exposures will jointly contribute to *NR3C1* methylation signatures at birth, and (3) *NR3C1* signatures at birth will be associated with infant outcomes. Infant outcomes considered included gestational age at delivery, birth weight, birth length, head circumference, and cephalization index (a ratio of head circumference to body weight; a marker of asymmetric intrauterine growth) [36]. We test these hypotheses using data from a prospective birth cohort study while controlling for a range of maternal and infant factors. 

## 2. Materials and Methods

### 2.1. Study Population

**Figure 1:**
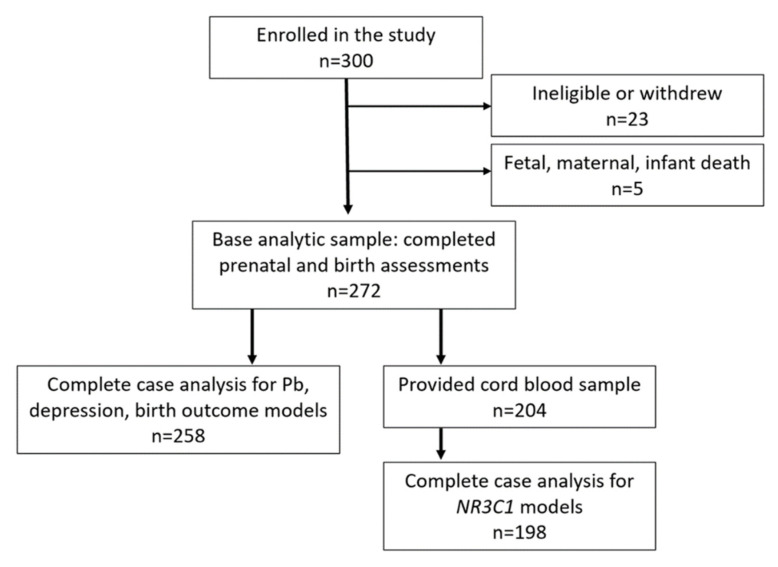
Albany Infant and Mother Study participation.

Participants were part of the Albany Infant and Mother Study (AIMS), a prospective observational cohort study of pregnant women and their infants born at the Albany Medical Center (Albany, New York, NY, USA) [37]. English-speaking women, 18–40 years old, with singleton pregnancies were eligible to participate. Women enrolled on average at 27 weeks gestation at an Albany Medical Center outpatient obstetrics clinic. At the prenatal enrollment visit, participants completed questionnaires that assessed demographic factors, health histories, behaviors, psychosocial factors, depressive symptoms, and environmental exposures. Maternal toenail samples were collected at the enrollment visit and assayed for metals concentrations. An umbilical cord blood sample was collected at birth by trained delivery room clinicians and assayed for epigenetic information. Following the birth, study physicians conducted a structured medical record review to obtain clinical information on maternal health, delivery, and infant characteristics, including birth outcome information. Three-hundred mother–infant pairs enrolled, with *n* = 272 eligible participants completing the prenatal and birth assessments, of whom *n* = 204 provided an umbilical cord blood sample (Figure 1). As 25% of the base analytic sample was missing umbilical cord blood samples, we conducted two sets of complete case analyses to address study hypotheses: (1) those with complete information on depression, Pb, infant outcome and covariate data (*n* = 258), and (2) those with depression, Pb, infant outcome, covariate, and epigenetic data (*n* = 198). Protocols and informed consent documents were approved by Institutional Review Boards at Albany Medical Center and the University at Albany State University of New York.

### 2.2. Measures

Lead (Pb). Pb exposure was measured from maternal toenail clippings collected at the prenatal enrollment visit. Previous research has shown that metals concentrations in toenails characterize exposure over the previous 2 to 12 months [38], thus providing a summary measure of exposure from the pregnancy and perinatal periods. Samples were stored at room temperature and nails from both big toes were assayed for metals concentrations. Samples were analyzed with inductively coupled plasma mass spectrometry in the Trace Element Analysis (TEA) facility at Dartmouth College. The TEA core operates an Element2 high-resolution ICP-MS (Thermon Finnegan, Bremen, Germany) and a quadrupole collision cell 7500c ORS ICP-MS (Agilent, Santa, Clara, CA, USA). Before ICP-MS analysis, nail clippings were washed to remove all external contamination without extracting metals from inside the nails. Nails were then dried, subjected to microwave digestion, and metals analysis was conducted by collision cell ICP-MS. Detection limits are on the order of 0.02 µg/g nail. All sample preparations and analyses were carried out in a trace-metal clean HEPA-filtered-air environment. Analytical blanks and potential instrumental drifts were monitored, and instrument standardization and reproducibility were performed with NIST traceable standards and Certified Standard Reference Materials. All laboratory quality control/quality assurance (QA) procedures were strictly followed to assure analytical accuracy. QA procedures included a 10% re-analysis of masked, replicate samples of toenail clippings. If the coefficient of variability exceeded 15%, the batch was re-analyzed. All laboratory personnel were blinded to participant information. Pb concentrations (range: 0.02 to 12.67 µg/g) were dichotomized with the top tertile of the distribution signifying higher levels of exposure (top tertile range: 0.71–12.67 µg/g). This dichotomous Pb variable was used in analysis. 

Depression. Prenatal depression was measured with the Edinburgh Postnatal Depression Scale (EPDS; α = 0.87), a self-report scale that has been validated for use among pregnant and postpartum populations and focuses on the cognitive and affective features of depression rather than somatic complaints [39]. The EPDS assesses the intensity of depressive symptoms in the past week. Select items were reverse scored and responses were summed to obtain a total depressive symptoms score (reported range in this sample: 0–27), with higher scores indicating more depressive symptoms. Total summary scores and a binary score indicating clinically significant depressive symptoms (scores > 12.5 considered depressed) were examined in analysis. 

Infant Birth Outcomes. Anthropometric measurements of the newborn were conducted in the delivery room following the birth by trained clinical staff using standard protocols and instrumentation. Information on gestational age at birth (weeks), birth weight (grams), birth length (centimeters), head circumference (centimeters) were abstracted from medical records. In addition, a cephalization index ((head circumference cm/birth weight g) × 100)) was derived. This index reflects the degree of asymmetric intrauterine growth (e.g., larger heads proportional to body size), with higher scores indicating potential brain sparing during gestation and offspring neurodevelopmental vulnerability [36,40]. All infant outcomes were treated continuously in analysis.

*NR3C1* DNA Methylation. DNA from umbilical cord blood samples was bisulfite converted, randomly allocated to different plates and chips, and assayed for epigenome wide DNA methylation using the Illumina EPIC Infinium array (Illumina, San Diego, CA, USA) [41]. The EPIC array quantifies DNA methylation at over 850,000 CpG sites in enhancer regions, gene bodies, promoters, and CpG islands and has over 90% of the original Infinium Methylation450 BeadChip content. The EPIC array was performed at the USC Molecular Genomics Data Production Core. Methylation data were preprocessed in R for quality control, background correction, normalization, type 1 and II probe scaling, and batch adjustment following established protocols [42]. Specifically, DNA methylation was extracted from raw files using the *minfi* R package (http://bioconductor.org/packages/release/bioc/html/minfi.html; accessed on 5 December 2018). Functional normalization was conducted to remove poor quality probes that fell below the detection limit (*p* < 0.01) and data were adjusted for type 1 and type 2 probe variation via the Beta Mixture Quantile Dilation (BMIQ) function of the wateRmelon package (https://www.bioconductor.org/packages/release/bioc/html/wateRmelon.html; accessed on 5 December 2018), batch effects were adjusted with the ComBAT (https://rdrr.io/bioc/sva/man/ComBat.html; accessed on 5 December 2018). In addition, β values (representing a ratio of methylated versus unmethylated DNA at each CpG site) were adjusted for cell-type heterogeneity via *estimateCellCounts*. This analysis focused on the 85 *NR3C1* CpG sites.

Covariates. Covariates were selected a priori based on their potential to confound the associations [43] and included demographic, maternal health, and infant factors. Demographics included self-reported maternal age, race/ethnicity (white not-Hispanic versus Black/Hispanic/other) and education attainment (high school degree or less versus more than high school). Infant sex was abstracted from medical records (male/female). Maternal health factors included pre-pregnancy body mass index (weight in kilograms/height in meters [2]; calculated from self-reported pre-pregnancy height and weight provided at the enrollment visit), self-reported smoking during pregnancy (yes/no), and diet during pregnancy. Diet as assessed in pregnancy with a 25-item Food Frequency Questionnaire [44,45]; a “Western” diet sum score reflected the frequency of consumption of foods from Western categories (e.g., red meats, processed meats, refined grains, high-fat dairy products, potatoes, and sugar-sweetened beverages). Delivery factors abstracted from medical records included parity (nulliparous or not) and mode of delivery (vaginal, c-section). 

### 2.3. Analytic Plan

First, using the pre-processed and cell type-corrected methylation data, we summarized DNA methylation across the 85 *NR3C1* CpGs via factor analysis, which is a data reduction strategy that allows for the distillation of a large number of observed variables into a smaller number of latent variables that can represent methylation variability across the gene while also preventing type I error. This approach is useful for reducing the high-dimensionality in methylation, gene-expression, and microarray datasets [46,47,48]. We conducted an exploratory factor analysis with an oblimin rotation, which allowed factor solutions to be correlated, followed by a parallel analysis to determine the number of factors to retain. Parallel analysis used a Monte Carlo simulation where a random simulated data set is generated and eigenvalues from the simulated data are compared to those generated from the actual data; the number of factors where the eigenvalue in the simulated data is higher than what was observed in the actual data is considered significant and should be retained [49,50]. For each retained factor, continuous factor scores, which represent the participant’s relative standing on each latent factor, was used in subsequent analyses related to maternal depression, Pb, and infant outcomes. 

Distributions of continuous study variables were examined through visual inspections of plots, and skewness and kurtosis statistics. Next, those with and without epigenetic information were compared according to demographics, Pb, and depression via independent *t*-tests and chi-square tests. Then, descriptive statistics were generated for all study variables and bivariate associations were assessed via Pearson’s correlations. To test the hypothesis that depression during pregnancy will potentiate the effect of prenatal Pb exposure and lead to worse infant outcomes than either exposure independently, adjusted linear regression models testing the main effects and multiplicative interaction between prenatal Pb and depression exposures were fit for a panel of birth outcomes: gestational age, birth weight, length, head circumference, cephalization index. Similarly, to test the hypothesis that gestational Pb and depression exposures will jointly contribute to *NR3C1* methylation signatures at birth, adjusted linear regression models tested the main effects and multiplicative interaction between Pb and depression for each *NR3C1* methylation factor as outcomes. Finally, we considered whether the *NR3C1* methylation factors were associated with infant outcomes via adjusted linear regressions. We did not formally test *NR3C1* mediation due to the lower sample size for the epigenetic data. Depression was considered continuously and dichotomously in regression analyses. A backward elimination approach was used in all regression models to select covariates for adjustment: we began with a saturated model, identified the covariate with the highest *p*-value, eliminated it, and refit the model. This process was repeated until all variables remaining in the equation were significant at *p* < 0.05. Akaike information criterion (AIC) was used to determine goodness-of-fit. Statistical significance was determined by *p*-values < 0.05 and 95% confidence limits. Marginal significance was determined by *p* < 0.10. Analyses were performed using SAS software version 9.4 (SAS Institute Inc., Cary, NC, USA).

## 3. Results

The DNA methylation factor analysis and parallel analysis confirmed that six *NR3C1* factors should be retained with fit statistics indicating acceptable model fit (REMSA = 0.057). Supplemental Table S1 lists the CpGs, genomic location, methylation extent, and factor loading according to each of the 6 *NR3C1* factors. Factors 1, 2, and 6 generally reflected high methylation extents in the gene transcription start sites (TSS), 3′ and 5′ untranslated regions (UTR), and the gene body. Factor 3 generally reflected CpGs with low methylation extents in 5′UTR and TSS sites. Factors 4 and 5 generally reflected moderate levels of methylation in TSS, 5′UTR, and gene body regions. The proportion of variance explained in methylation by factors 1 through 6 was 0.36, 0.24, 0.12, 0.08, 0.07, and 0.13, respectively. 

Those missing DNA methylation information were more likely to be racial/ethnic minorities and have lower levels of education (all *p* < 0.05; data not shown); no differences by depression or Pb exposures according to available DNA methylation data were found (all *p* > 0.05). Participant characteristics are listed in Table 1. Women were on average 28.6 years old (range 18–40), 47% were from a racial/ethnic minority group, and 36% reported lower levels of education. The average Pb concentration among the full sample was 0.08 µg/g, with the average Pb concentration among the highest tertile as 1.85 µg/g (range 0.72–12.67 µg/g). The mean depressive symptoms score was 8.8, and 23% met the clinical threshold for depression. Infants were mostly term pregnancies, and of average birth size.

**Figure 2:**
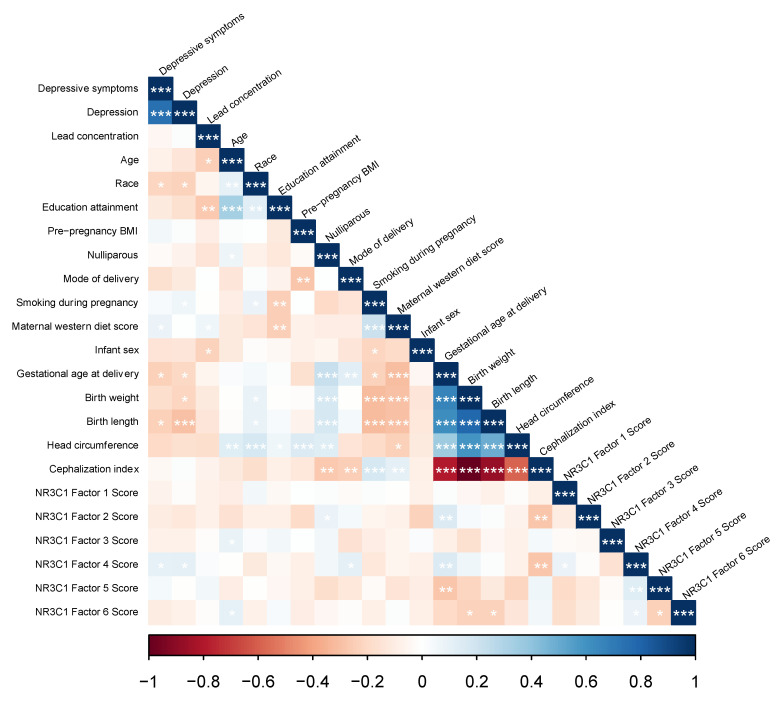
Pearson’s correlations among study variables. * *p* < 0.05, ** *p* < 0.01, *** *p* < 0.001.

Figure 2 shows correlations among all study variables. Depressive symptoms were negatively associated with gestational age and birth length. Pb was negatively correlated with birth outcomes, though correlations were not statistically significant. Depression and Pb were not correlated with each other. All covariates were significantly correlated with maternal depression, Pb, and/or birth outcomes. Specifically, higher levels of maternal depression was significantly correlated racial/ethnic minority status, smoking during pregnancy, higher Western diet score, earlier gestational age at delivery, lower birth weight, and shorter birth length. Higher levels of Pb was significantly correlated with younger maternal age, lower maternal education attainment, higher Western diet score, and male infant sex. Pre-pregnancy BMI, parity, mode of delivery, were not correlated with depression or Pb, but were correlated with the infant birth outcomes. *NR3C1* factor scores were correlated with some infant outcomes and study variables. Factors 2 and 4 were each positively correlated with gestational age and negatively correlated with cephalization index. Factor 5 was negatively correlated with gestational age. Factor 6 was negatively associated with birth weight and birth length. Factor 4 was correlated with maternal depression. Factors 3 and 6 were associated with maternal age; factor 2 was correlated with parity; factor 4 was correlated with delivery model. *NR3C1* factor scores were not significantly correlated with other study variables. Factor 1 was not significantly correlated with infant outcomes or other covariates. 

**Figure 3:**
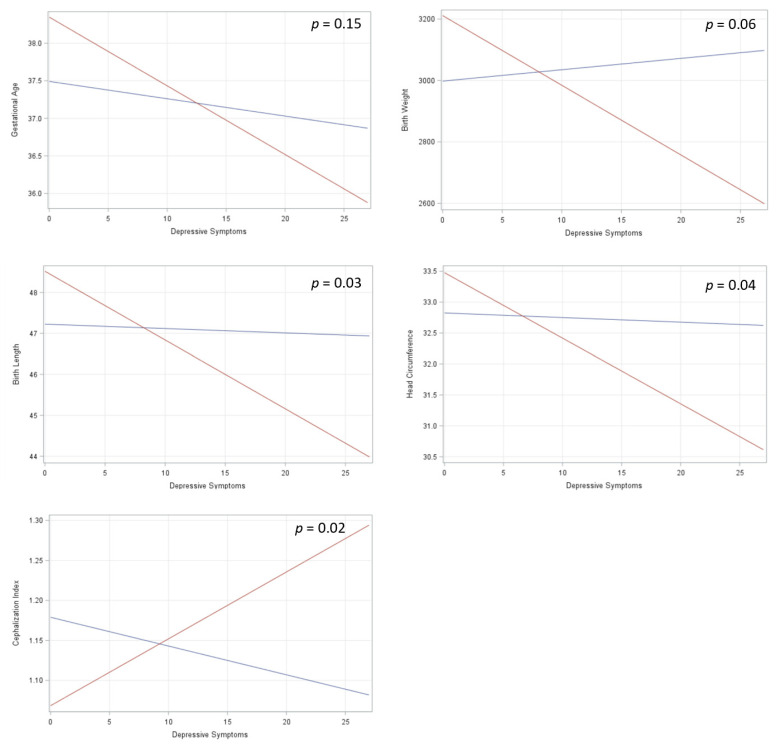
Interaction effects for the joint contribution of prenatal Pb exposure and maternal depression during pregnancy in relation to birth outcomes. Red lines depict slopes for those with high Pb exposure, and blue lines depict slopes for those with lower prenatal Pb exposure. *p*-values for the interaction effect for Pb and depressive symptoms from each outcome are listed in their respective graphs. Models (final covariates determined via backward elimination): *Y_GA_* = *β_0_* + *β_Pb_* + *β_Depression_* + β_Pb*Depression_ + *β_Parity_* + *β_Smoker_* + *β_DeliveryMode_*; *Y_BW_* = *β_0_* + *β_Pb_* + *β_Depression_* + β_Pb*Depression_ + *β_Race_* + *β_Parity_* + *β_Smoker_* + *β_Diet_* *+* *β_InfantSex_*; *Y_BL_* = *β_0_* *+* *β_Pb_* *+* *β_Depression_* *+* β_Pb*Depression_ *+* *β_Race_* *+* *β_Parity_* *+* *β_Smoker_* *+* *β_Diet_*; *Y_HC_* = *β_0_* *+* *β_Pb_* *+* *β_Depression_* *+* β_Pb*Depression_ *+* *β_Race_* *+* *β_Age_* *+* *β_Parity_* *+* *β_BMI_ +* *β_Smoker_* *+* *β_DeliveryMode_*; *Y_Ceph_* = *β_0_* *+* *β_Pb_* *+* *β_Depression_* *+* β_Pb*Depression_ *+* *β_Parity_* *+* *β_Smoker_* *+* *β_DeliveryMode_*.

The results of the multivariable linear regression models testing the interaction between prenatal Pb and depression exposures for birth outcomes are displayed in Figure 3. There was a significant interaction between prenatal Pb and depressive symptoms for head circumference, birth length, and cephalization index. A marginally significant interaction was found for birth weight. An interaction trend was evident for gestational age, but was not significant (*p* = 0.15). Supplemental Table S2 shows the models for the main effects of depression and Pb for each birth outcome, as well as the interaction effects depicted in Figure 3. 

Table 2 lists the multivariable linear regression models for the associations between maternal Pb, depression, with *NR3C1* factor scores as separate outcomes. Marginally significant interactions between Pb and depression were observed for *NR3C1* factors 1 and 2, indicating that higher levels of both exposures may be jointly influencing methylation extents at the CpGs loading on these factors.

Table 3 lists the multivariable linear regression models testing the association between each *NR3C1* DNA methylation factor score with infant outcomes. Differential patterning was observed for *NR3C1* factor scores and infant outcomes. Higher *NR3C1* scores for factors 2 and 4 were significantly associated with longer gestations, less cephalization, and marginally higher birth weight (factor 4). Higher *NR3C1* scores for factors 5 and 6 were associated with shorter gestations, lower birth weights and increased cephalization. *NR3C1* factors 1 and 3 were not significantly associated with any infant outcome.

## 4. Discussion

In this study, we found that depression during pregnancy and prenatal Pb exposure synergistically contributed to earlier gestational age and smaller infant body size at birth. Prenatal depression and Pb exposures also jointly contributed to some gestational *NR3C1* methylation signatures; *NR3C1* methylation signatures were in turn associated with infant outcomes. Our findings are consistent with a developmental origins of health and disease framework and illustrate that adverse environmental and psychosocial exposures occurring during gestation may synergistically affect birth outcomes and epigenetic mechanisms. Moreover, our study suggests that gestational modulation of an HPA axis-related gene could possibly represent a shared biologic pathway linking Pb and depression exposures to infant outcomes. Our findings are particularly noteworthy as we controlled for important sociodemographic, maternal health, delivery, and infant factors that could potentially confound the associations.

We found support for our hypothesis that maternal depression in conjunction with prenatal Pb exposure would lead to worse birth outcomes than either exposure independently. Indeed, the main effects of depression and Pb were largely null; it was only when considering the accumulated impact of both exposures did a deleterious association emerge, which was consistently observed across all birth outcomes. This finding is congruent to what related perinatal work has reported when considering synergies between prenatal air pollution and socioeconomic exposures [22], and builds the evidence base to show similar patterning in birth outcome associations for Pb and depression exposures as well. Being born small or early are important public health concerns as they can indicate neonatal morbidity and mortality risk, as well as help to characterize health risk across the life course [1]. From a developmental origins of health and disease perspective, birth size and gestational age can also indicate the quality of the intrauterine environment and life-long programming effects [1]. Psychosocial and environmental toxicant exposures can commonly co-occur, and in the context of gestational programming and developmental plasticity, may be simultaneously working to shape developing body systems and the future health risk for children [1,7]. Our findings underscore this possibility. However, as we were unable to assess whether health risk continued past the neonatal period, we encourage future work to incorporate a longer follow up period to test whether health risk stemming from gestational environmental and psychosocial exposures endures or remediates over time.

We found some evidence to suggest *NR3C1* methylation may be likewise influenced by both gestational Pb and depression exposures, which in turn may influence infant outcomes. Specifically, we found that *NR3C1* methylation signatures among the two primary factors (factors 1 and 2) were the most sensitive to depression and Pb exposures, though associations were marginally significant. These factors represented largest proportion of the variability in *NR3C1* methylation (60%), and CpGs that loaded on these factors generally had high degrees of methylation extents across genomic regions, suggesting potential dysregulation of *NR3C1*. While *NR3C1* factor 1 score was not associated with infant outcomes, *NR3C1* factor 2 scores were associated with gestational age and neonate cephalization. These findings are congruent with some prior work that has shown that *NR3C1* methylation to be independently associated with prenatal depression exposures [35], neurotoxic metals [34], and infant outcomes [31,33,46]. Our findings bridge these independent literatures and provides some initial evidence that *NR3C1* methylation extents (particularly among CpGs loading on factor 2) could potentially link gestational metal and psychosocial exposures to infant phenotype. We encourage future work with larger samples to replicate these findings and conduct formal tests of mediation to test this possibility explicitly.

While *NR3C1* factors 3–6 were not jointly influenced by prenatal depression and Pb exposures and represent a smaller degree of *NR3C1* variability, these factors were each associated with infant outcomes. For example, higher loadings on *NR3C1* factor scores 5 and 6 were associated with shorter gestations and smaller infant size at birth whereas higher loadings on factor 4 was related to longer gestations and less restricted fetal growth. These associations were maintained when controlling for key contributors of gestational methylation signatures and birth outcomes, including smoking during pregnancy. However, none of the prenatal psychosocial, environmental, or maternal behavioral exposures we considered correlated with factor 3–6 methylation scores. Thus, while we observed a signal between gestational *NR3C1* methylation extent and birth outcomes (as is consistent with the literature), we could not discern what exposures contributed to *NR3C1* methylation extents for the CpGs loading on factors 3–6 in this analysis. Future work should consider the accumulated impacts and differential contributions of a broader set of toxicant and psychosocial exposures than was considered here in relation to *NR3C1* methylation. For example, a weighted quantile sum regression approach, which involves deriving an index of multiple correlated predictors (e.g., multiple metals, other toxicants, maternal life course psychosocial factors, maternal behaviors, and sociodemographics) that are weighted according to their strength of association with an outcome [51] could help elucidate what combination of gestational exposures, as well as their relative contribution, to *NR3C1* methylation. 

This study has some limitations. First, the sample size was moderate and 25% of participants did not have available DNA methylation information. Those missing methylation information were more socially disadvantaged compared to those included in analysis, which could have constrained variability in the exposure and outcomes and lead to underestimated associations. Moreover, this missing data disallowed formal tests of mediation. We encourage future work with larger samples to formally test *NR3C1* as a mediator linking prenatal toxicant and psychosocial exposures with birth outcomes explicitly. In addition, we used infant body size measures that were recorded at birth and as such may be imprecise; head circumference and body length measures assessed in the postpartum period would have been superior but were not available. Additionally, while the EPDS has been validated for use among pregnant and postpartum populations, it is a self-report and thus may be inaccurate. Additionally, information on alcohol intake during pregnancy, and pregnancy morbidity were not included in analysis and thus residual confounding by these factors may remain. Finally, we did not measure gene expression and thus can not conclude that the variability observed in methylation signatures equated to differences in gene expression. These limitations notwithstanding, this study also has several strengths. It is among the first to consider the joint contribution of prenatal exposure to depression and Pb in relation to fetal growth and to gestational DNA methylation to *NR3C1* DNA methylation. We used a multimodal prospective design that integrated information from biologic samples, validated questionnaires, and hospital medical records. We implemented a novel analytic strategy to reduce the dimensionality of the *NR3C1* methylation information which also reduces type I error. Moreover, we rigorously controlled for confounding by considering several demographic, maternal health, dietary, delivery, and infant characteristics in the multivariate models. 

## 5. Conclusions

Researchers are increasingly considering the accumulated impact of social and environmental exposures to explain health and health disparities. Our study adds to this emergent area of research and shows such joint effects are evident at the earliest time in life, and can influence molecular markers of HPA axis function, which in turn may be laying the foundation for health risk for a lifetime. Our findings are particularly noteworthy as depression and Pb exposures are common in the general population, and gestation is a life phase characterized by tremendous biologic plasticity that is amenable to public health intervention. Programs and policies that address environmental and psychosocial risks simultaneously during pregnancy may be better positioned to promote maternal and child health than traditional initiatives dedicated to mitigating one type of exposure. We encourage future research and policy work consider the joint influence of social and environmental factors in order to more effectively address the health consequences of these interconnected exposures. 

## Figures and Tables

**Table 1:** Participant characteristics (*n* = 258).

Characteristic	% or Mean (SD)
Depression during pregnancy	
Depressive symptoms score	8.81 (5.50)
Depression, yes	23.08
Depression, no	76.92
Lead exposure during pregnancy	
Lead concentrations, µg/g	0.80 (1.28)
High tertile, µg/g	1.85 (1.86)
Lower/Middle tertiles, µg/g	0.30 (0.19)
Maternal characteristics	
Age, years	28.56 (5.48)
Race, Black/Hispanic/Other	46.54
Race, White/Not Hispanic	53.46
Education attainment, high school or less	35.77
Education attainment, more than high school	64.23
Pre-pregnancy BMI	28.82 (8.49)
Nulliparous, yes	23.85
Nulliparous, no	76.15
Mode of delivery, c-section	33.85
Mode of delivery, vaginal	66.15
Smoking during pregnancy, yes	13.85
Smoking during pregnancy, no	86.15
Maternal Western diet score	39.81 (15.25)
Infant characteristics	
Infant sex, male	50.77
Infant sex, female	49.23
Gestational age at delivery, weeks	38.8 (2.14)
Birth weight, grams	3247.83 (639.27)
Birth length, centimeters	48.68 (3.36)
Head circumference, centimeters	33.43 (2.16)
Cephalization index	1.07 (0.23)

**Table 2:** Multivariable linear regression models testing the joint contribution of depression and Pb exposure during pregnancy on *NR3C1* DNA methylation factor scores.

	*NR3C1* Factor 1	*NR3C1* Factor 2	*NR3C1* Factor 3	*NR3C1* Factor 4	*NR3C1* Factor 5	*NR3C1* Factor 6
Predictor	*β (SE)*	*β (SE)*	*β (SE)*	*β (SE)*	*β (SE)*	*β (SE)*
Depression	0.33 (0.21) [−0.07, 0.74]	0.11 (0.21) [−0.30, 0.52]	−0.07 (0.20) [−0.46, 0.33]	0.04 (0.18) * [0.04, 0.76]	0.06 (0.19) [−0.31, 0.42]	0.07 (0.19) [−0.30, 0.44]
Pb	0.13 (0.17) [−0.21, 0.47]	0.14 (0.17) [−0.20, 0.52]	0.12 (0.17) [−0.22, 0.45]	0.16 (0.15) [−0.14, 0.46]	0.18 (0.16) [−0.13, 0.49]	0.22 (0.16) [−0.10, 0.54]
Depression × Pb	−0.58 (0.34) ^+^ [−1.25, 0.10]	−0.65 (0.35) ^+^ [−1.33, 0.03]	0.21 (0.34) [−0.45, 0.87]	−0.10 (0.30) [−0.70, 0.50]	0.17 (0.31) [−0.44, 0.78]	−0.20 (0.31) [−0.82, 0.42]

Top cell entries are β(SE); Bottom cell entries are [95% confidence limits]. ^+^ *p* < 0.10, * *p* < 0.01. Models (final covariates determined via backward elimination): Y_NR3C1Factor1_ = β_0_ + β_Pb_ + β_Depression_ + β_Pb*Depression_; Y_NR3C1Factor2_ = β_0_ + β_Pb_ + β_Depression_ + β_Pb*Depression_ + β_Parity_ + β_InfantSex_; Y_NR3C1Factor3_ = β_0_ + β_Pb_ + β_Depression_ + β_Pb*Depression_ + β_Age_; Y_NR3C1Factor4_ = β_0_ + β_Pb_ + β_Depression_ + β_Pb*Depression_ + β_DeliveryMode_; Y_NR3C1Factor5_ = β_0_ + β_Pb_ + β_Depression_ + β_Pb*Depression_; Y_NR3C1Factor6_ = β_0_ + β_Pb_ + β_Depression_ + β_Pb*Depression_ + β_Age_.

**Table 3:** Multivariable linear regression models testing the association between each NR3C1 DNA methylation factor score with infant outcomes ^.

Predictor	Gestational Age	Birth Weight	Birth Length	Head Circ.	Cephalization
*NR3C1* factor 1	0.11 (0.12) [−0.14, 0.35]	18.25 (42.08) [−64.80, 101.23]	0.01 (0.21) [−0.40, 0.42]	0.01 (0.13) [−0.26, 0.27]	−0.01 (0.01) [−0.03, 0.02]
*NR3C1* factor 2	0.32 (0.12) ** [0.08, 0.56]	38.44 (41.60) [−43.49, 120.68]	0.20 (0.20) [−0.20, 0.60]	0.08 (0.13) [−0.20, 0.32]	−0.04 (0.01) ** [−0.06, 0.01]
*NR3C1* factor 3	−0.09 (0.13) [−0.35, 0.16]	−60.28 (43.12) [−145.34, 24.79]	0.06 (0.21) [−0.36, 0.48]	−0.14 (0.14) [−0.41, 0.13]	0.03 (0.01) ^+^ [−0.001, 0.05]
*NR3C1* factor 4	0.38 (0.13) ** [0.12, 0.65]	80.14 (46.51) ^+^ [−11.56, 171.91]	0.26 (0.23) [−0.19, 0.71]	−0.05 (0.15) [−0.34, 0.25]	−0.04 (0.02) * [−0.07, −0.01]
*NR3C1* factor 5	−0.34 (0.14) * [−0.60, −0.07]	−73.59 (46.37) [−165.35, 17.66]	−0.09 (0.23) [−0.54, 0.36]	−0.31 (0.15) * [−0.60, −0.02]	0.03 (0.02) ^+^ [−0.004, 0.06]
*NR3C1* factor 6	−0.29 (0.13) * [−0.55, −0.03]	−101.13 (45.15) * [−190.04, −12.00]	−0.50 (0.22) * [−0.94, −0.06]	−0.17 (0.15) [−0.46, 0.12]	0.03 (0.02) ^+^ [−0.004, 0.06]

^^^ Top cell entries are β(SE); bottom cell entries are [95% confidence limits]. Models tested the association between each *NR3C1* factor score and infant outcomes independently; models did not include other factor scores as covariates. Final covariates determined via backward elimination. ^+^ *p* < 0.10, * *p* < 0.05, ** *p* < 0.01.

## Data Availability

The data presented in this study are available on request from the corresponding author. The data are not publicly available in order to maintain participant confidentiality.

## Supplementary Materials

The following are available online at https://www.mdpi.com/article/10.3390/ijerph182212169/s1, Table S1: Descriptive information for *NR3C1* factor scores; Table S2: Multivariable linear regression models testing the joint contribution of depressive symptoms and lead exposure during pregnancy on birth and infant outcomes.
