# Supplementary material for: Prenatal Lead and Depression Exposures Jointly Influence Birth Outcomes and NR3C1 DNA Methylation
Source: Int J Environ Res Public Health. 2021 Nov 19;18(22):12169. doi: 10.3390/ijerph182212169 (PMC8620070; doi:10.3390/ijerph182212169)
Supplement: Supplementary file 1 [file ijerph-18-12169-s001.zip › ijerph-1447364-supplementary.pdf]

Supplemental Table S1. Descriptive information for *NR3C1* methylation factor scores

CpG	Location	% Methylated	Factor	Factor Loading
cg14621978	Body	0.96 ± 0.02	1	0.91
cg12466613	TSS1500	0.97 ± 0.01	1	0.83
cg23273257	3'UTR	0.98 ± 0.01	1	0.82
cg19176661	Body	0.96 ± 0.02	1	0.80
cg14438279	5'UTR	0.95 ± 0.02	1	0.79
cg00407401	Body	0.93 ± 0.03	1	0.77
cg05900547	Body	0.90 ± 0.05	1	0.75
cg05900547	Body	0.90 ± 0.05	1	0.75
cg07528216	5'UTR	0.97 ± 0.01	1	0.74
cg26081259	3'UTR	0.97 ± 0.01	1	0.72
cg16535116	Body	0.88 ± 0.04	1	0.71
cg27345592	5'UTR	0.97 ± 0.01	1	0.68
cg21979215	TSS1500	0.83 ± 0.07	1	0.63
cg07715663	Body	0.96 ± 0.03	1	0.58
cg19457823	Body	0.94 ± 0.04	1	0.54
cg24801588	Body	0.94 ± 0.02	1	0.54
cg00294552	5'UTR	0.91 ± 0.03	1	0.48
cg06613263	Body	0.93 ± 0.02	1	0.47
cg25708981	Body	0.88 ± 0.05	1	0.47
cg07589972	TSS1500	0.95 ± 0.01	1	0.42
cg01751279	5'UTR	0.93 ± 0.02	1	0.38
cg08845721	5'UTR	0.97 ± 0.01	1	0.36
cg25579735	5'UTR	0.98 ± 0.01	1	0.36
cg12888360	Body	0.53 ± 0.12	1	0.35
cg00629244	TSS200; 5'UTR	0.01 ± 0.002	1	-0.32
cg13648501	TSS1500; 5'UTR	0.03 ± 0.01	1	-0.37
cg06952416	5'UTR	0.07 ± 0.01	1	-0.43
cg14558428	TSS1500; 5'UTR	0.02 ± 0.002	1	-0.47
cg26720913	5'UTR; 1stExon	0.01 ± 0.005	1	-0.47
cg27122725	5'UTR	0.06 ± 0.01	1	-0.48
cg08818984	1st Exon; 5'UTR	0.02 ± 0.01	1	-0.52
cg03746860	Body	0.93 ± 0.07	2	0.91
cg16224829	5'UTR	0.86 ± 0.07	2	0.88
cg25535999	Body	0.95 ± 0.02	2	0.85
cg01294526	TSS1500	0.95 ± 0.03	2	0.84
cg12969488	5'UTR	0.79 ± 0.05	2	0.77
cg23430507	5'UTR	0.81 ± 0.04	2	0.67
cg20728768	Body	0.67 ± 0.10	2	0.56
cg16594263	Body	0.97 ± 0.01	2	0.45
cg07589972	TSS1500	0.95 ± 0.01	2	0.42
cg14438279	5'UTR	0.95 ± 0.02	2	0.41

cg24052866	Body	0.86 ± 0.04	2	0.41
cg25579735	5'UTR	0.98 ± 0.01	2	0.39
cg13764763	TSS1500; 5'UTR	0.95 ± 0.02	2	0.38
cg07528216	5'UTR	0.97 ± 0.01	2	0.33
cg12741214	Body	0.97 ± 0.01	2	0.32
cg23776787	5'UTR; 1stExon	0.02 ± 0.005	2	-0.38
cg08818984	1stExon; 5'UTR	0.02 ± 0.01	2	-0.56
cg03906910	5'UTR; 1stExon	0.15 ± 0.07	2	-0.75
cg15115787	Body	0.43 ± 0.12	2	-0.80
cg15910486	TSS1500; 5'UTR	0.02 ± 0.004	3	0.65
cg18019515	TSS200; 5'UTR	0.01 ± 0.001	3	0.52
cg14939152	TSS1500; 5'UTR	0.02 ± 0.002	3	0.51
cg17617527	5'UTR	0.02 ± 0.002	3	0.48
cg21209684	TSS1500; 5'UTR	0.02 ± 0.002	3	0.48
cg17860381	TSS1500; 5'UTR	0.01 ± 0.003	3	0.45
cg18068240	TSS1500; 5'UTR	0.01 ± 0.002	3	0.43
cg01967637	TSS1500; 5'UTR; 1st Exon	0.07 ± 0.03	3	0.41
cg04111177	TSS1500; 5'UTR	0.02 ± 0.006	3	0.41
cg10847032	TSS1500; 5'UTR	0.02 ± 0.004	3	0.40
cg14558428	TSS1500; 5'UTR	0.02 ± 0.002	3	0.40
cg18146873	5'UTR; 1st Exon	0.02 ± 0.004	3	0.38
cg16335926	TSS1500; 5'UTR	0.01 ± 0.002	3	0.35
cg18849621	TSS1500; 5'UTR	0.02 ± 0.003	3	0.34
cg06968181	TSS1500; 5'UTR	0.05 ± 0.01	3	0.31
cg15645634	TSS1500; 5'UTR	0.01 ± 0.002	3	0.31
cg11152298	TSS200; 5'UTR	0.02 ± 0.01	3	0.30
cg19645279	Body	0.69 ± 0.06	3	-0.44
cg17342132	Body	0.92 ± 0.04	4	0.81
cg22233604	Body	0.78 ± 0.05	4	0.79
cg18998365	5'UTR	0.59 ± 0.04	4	0.34
cg20728768	Body	0.67 ± 0.10	4	-0.34
cg15115787	Body	0.43 ± 0.12	4	-0.44
cg03857453	Body	0.97 ± 0.02	5	0.55
cg08695103	Body	0.74 ± 0.08	5	0.48
cg18718518	TSS1500; 5'UTR	0.75 ± 0.03	5	0.44
cg20728768	Body	0.67 ± 0.10	5	0.42
cg24052866	Body	0.86 ± 0.04	5	0.42
cg21702128	TSS1500; 5'UTR	0.05 ± 0.01	5	0.38
cg11152298	TSS200; 5'UTR	0.02 ± 0.01	5	0.34
cg07742588	5'UTR	0.84 ± 0.07	6	0.67
cg17349736	5'UTR	0.93 ± 0.02	6	0.67

cg00294552	5'UTR	0.91 ± 0.03	6	0.44
cg24801588	Body	0.94 ± 0.02	6	0.43
cg25708981	Body	0.88 ± 0.05	6	0.43
cg05483455	Body	0.98 ± 0.02	6	0.41
cg20598211	Body	0.96 ± 0.01	6	0.34
cg18068240	TSS1500; 5'UTR	0.01 ± 0.002	6	0.33
cg15645634	TSS1500; 5'UTR	0.01 ± 0.002	6	0.31
cg21979215	TSS1500	0.83 ± 0.07	6	0.31
cg16335926	TSS1500; 5'UTR	0.01 ± 0.002	6	-0.42
cg27122725	5'UTR	0.06 ± 0.01	6	-0.49

The proportion of variance explained in methylation by factors 1 through 6 was 0.36, 0.24, 0.12, 0.08, 0.07, and 0.13 respectively.

Supplemental Table S2. Multivariable linear regression models testing the joint contribution of depressive symptoms and lead exposure during pregnancy on birth and infant outcomes (n=258)^

	Model 1	Model 2	Model 3	Model 4
**Gestational Age**				
Depression	-0.05 (0.02)		-0.05 (0.02)	-0.02 (0.02)
	0.05		0.04	0.41
Lead		0.23 (0.27)	0.24 (0.27)	0.86 (0.03)
		0.39	0.37	0.10
Depression x Lead				-0.07 (0.05)
				0.15
**Birth Weight**				
Depression	-5.46 (6.84)		-5.43 (6.85)	3.70 (8.37)
	0.43		0.43	0.66
Lead		-22.78 (80.43)	-22.03 (80.49)	213.23 (8.37)
		0.78	0.78	0.15
Depression x Lead				-26.39 (14.03)
				0.06
**Birth Length**				
Depression	-0.07 (0.04)		-0.07 (0.04)	-0.01 (0.04)
	0.07		0.07	0.81
Lead		-0.12 (0.42)	-0.11 (0.42)	1.29 (0.78)
		0.77	0.79	0.10
Depression x Lead				-0.16 (0.07)
				0.03
**Head Circumference**				
Depression	-0.04 (0.02)		-0.04 (0.02)	-0.01 (0.03)
	0.07		0.07	0.79
Lead		-0.26 (0.27)	-0.24 (0.27)	0.65 (0.51)
		0.34	0.38	0.20
Depression x Lead				-0.10 (0.05)
				0.04
**Cephalization Index**				
Depression	0.001 (0.002)		0.001 (0.02)	-0.004 (0.003)
	0.83		0.83	0.24
Lead		-0.003 (0.03)	-0.003 (0.03)	-0.11 (0.05)
		0.91	0.91	0.04
Depression x Lead				0.01 (0.01)
				0.02

^Top cell entries are β (SE); bottom cell entry is the p-value for that model parameter. Depression is treated continuously.
